# Does diet and activity lead to difference in resting energy expenditure in obese women?

**DOI:** 10.1186/s12905-023-02480-3

**Published:** 2023-06-24

**Authors:** Zahra Taghadomi Masoumi, Hamideh Pishva

**Affiliations:** Department of cellular-Molecular Nutrition, School of Nutrition Sciences and Dietetics, Tehran University of Medical Sciences, Tehran, Iran

**Keywords:** Resting energy expenditure, Obesity, Dietary intake, Lipid, Thyroid hormones

## Abstract

**Background:**

Obesity is considered as a risk factor for metabolic and chronic diseases. Reduction in resting energy expenditure (REE) may increase risk of obesity. Our study was carried out to investigate dietary, biochemical, anthropometric and body composition parameters and physical activity in obese women with normal and low resting energy expenditure.

**Methods:**

A total forty nine subjects (women, 30-50 years old) were enrolled and divided into three groups. Anthropometric, body composition parameters, resting energy expenditure, Fasting blood lipid profile, dietary intake and physical activity were measured.

**Results:**

Although, fat mass and fat-free mass were significantly increased in obese groups, there was no significant difference in body composition between two obese groups (p-value = 0.10, 0.27). Measured resting energy expenditure was significantly decreased in obese with low REE compare to other groups (p-value < 0.001). There was no significant difference in energy intake and macronutrients between groups. There was a significant difference in T3 between obese subjects with low REE compared to obese group with normal REE (p-value < 0.001). There was no significant difference in lipid profile between two obese groups. Also there was a significant difference in LDL, cholesterol and triacylglycerol between obese subjects with low REE compared to normal weight group. Moreover, there was a significant difference in cholesterol and triacylglycerol between obese subjects with normal REE compared to normal weight group. Our finding showed there was no significant difference in physical activity between three groups.

**Conclusions:**

Dietary intake and physical activity may relate to metabolism and energy expenditure. It is interesting that in some obese people resting energy expenditure was much lower compared to other obese people; however, there was no significant difference in their body composition, age, sex, dietary intake, lipid profile and physical activity. Thus it should investigate the role of other factors involved in different REE in subjects with obesity.

## Background

Obesity is recognized as the most prevalent metabolic disease. World health organization (WHO) has already declared that obesity is a global epidemic in which constitutes one of the biggest current health problems. It has been predicted that by 2030 approximately 60% of world’s population will be overweight and obese [1]. According to 2004-05 National Institutes of Health (NHS) survey, morbidly obese people were more prevalent among women in all age groups [2]. Low resting energy expenditure (REE) is considered as a risk factor for weight gain leading to Obesity [3]. It was shown that detecting impairment in REE may predispose an individual to weight gain is difficult once that subject is obese [4]. Some studies showed, since obese subjects have a greater amount of fat-free mass (FFM) as well as fat mass (FM) than normal weight subjects, therefore REE is typically higher in obesity [3]. Other studies showed REE in obese people was similar to normal weight people [4]. Recent studies showed that some obese subjects had a low resting energy expenditure and they defined these people as a hypometabolim [4]. Some mechanisms may involve in decreasing REE in obese subjects; mitochondrial dysfunction, mitochondrial degeneration and down-regulation of metabolism genes occurred in these people [5]. There is limited data available on dietary, biochemical and metabolic factors associated with obesity in hypometabolism obese people. Our study was carried out to investigate dietary, biochemical, anthropometric and body composition parameters and physical activity in obese women with normal and low resting energy expenditure.

## Subjects and methods

### Subjects

A total forty nine subjects (women, 30-50 years old), were enrolled and divided into three groups: 16 subjects with BMI > 30 and low resting energy expenditure, 17 subjects with BMI > 30 and normal resting energy expenditure, and 16 subjects with BMI < 25 and normal resting energy expenditure as a control group. Subjects with measured resting energy expenditure less than 20% of their predicted resting energy expenditure were defined as low resting energy expenditure [4]. They were selected according to the defined inclusion criteria: age 30-50 years, no medical history of diabetes, coronary, thyroid or other hormonal diseases, no medical history of endocrine abnormalities that related to secondary causes of obesity or gastrointestinal surgery for weight lost, no use of medications or treatments effective on their REE, no alcohol, smoking or drug abuse, no use of supplementary vitamins, not being pregnant, lactating or in menopause. When subjects do not have interest, they were excluded.

We calculated the sample size as fallow:

$${Z}_{1-\frac{\alpha }{2}}=1.96\,\,\,\,\,{\alpha }=0.05\,\,\,\,\,1-{\beta }=0.80\,\,\,\,\,{Z}_{1-\beta =}0.84$$

$$\eqalign {\varvec{n}& ={\left(\frac{{Z}_{1-\frac{\alpha }{2}}+{Z}_{1-\beta }\sqrt{1-{r}^{2}}}{r}\right)}^{2} \cr & +2={ \left(\frac{1.96+0.84\times \sqrt{1-{0.8}^{2}}}{0.8}\right)}^{2}+2=11.5}$$

According to the equation above, we calculated the sample size to be nearly 12 subjects in each group and selected more topics for outstanding accuracy.

All procedures performed were approved by the ethics committee of Tehran University of Medical Sciences (TUMS). All participants were informed of the nature of the study and gave a written informed consent.

### Anthropometric measurements

Height and weight were measured by a Seca scale (Germany) with light clothing and no shoes on. Body mass index (BMI) was then calculated. Waist circumference was measured with a flexible tape midway between the lowest rib and the iliac crest. The hip circumference was measured at the widest part of the gluteal region.

### Resting metabolic rate and body composition measurements

Resting Metabolic Rate (RMR) was measured by means of the Meta Check™ (Korr Medical Technologies, Salt Lake City, Utah), an instrument designed to measure RMR using indirect calorimetry.

All measurements were carried out while the participants were sitting in a thermo-neutral room after a 12-h overnight fast and having refrained from caffeine, nicotine, or exercise for at least 4 h. The device measures oxygen consumption through breath sampling over a duration of 20 min and then calculated REE and respiratory quotient (RQ).

Predicted resting energy expenditure for each subject was obtained using the Harris-Benedict Eq. [6].

Body composition was measured by bioelectrical impedance with the RJL Systems, Model Quantum II, a four terminal single frequency (800 µA at 50 Khz) impedance plethysmograph (RJL Systems, Clinton Twp, MI, USA) with an internal calibration system. Measurements were performed on all subjects by professional nutritionists.

All methods were carried out in accordance with relevant guidelines.

### Laboratory analyses

Blood sample was taken after 12 h of overnight fasting. Thyroid hormones (total T_3_ and T_4_) and Thyroid Stimulating Hormone (TSH) were measured by ELISA Kit (Padginteb Co. under license of ZellBio GmbH, Germany). Serum total cholesterol (TC), triacylglycerol (TAG) and Serum high density lipoprotein (HDL) levels were analysed by Assay Kit (PAD-CHO for TC, PAD-TG for TAG and PAD-HDL for HDL, Zell Bio GmbH, Germany). The serum low-density lipoprotein cholesterol (LDL-C) was calculated using the Friedewald formula: LDL = TC - HDL - TG/5.0 (mg/dL).

### Dietary intake and physical activity assessment

Dietary intake was assessed using 3-days food records. Subjects were asked to record the type and amount of food and beverage consumed for two consecutive weekdays and one weekend day, using standard household measures (cups, tablespoons, etc.). Trained interviewers reviewed the records along with the subject to clarify servings, recipes, and forgotten foods. Food intake data was analysed and energy and nutrient intake calculated using the Nutritionist4 (Hearst Corp. San Bruno, CA) Analysis software as modified for the Iran population.

The global physical activity questionnaire (GPAQ) is a standardized questionnaire developed by WHO as a physical activity surveillance instrument. We used our translation of the second version of GPAQ in the survey. This standardized questionnaire is consisted of 16 questions in duration and intensity (vigorous and moderate) of physical activity which is categorized in to three parts including work, transportation and recreation activities.

### Statistical analyses

The one-sample Kolmogorov-Smirnov test was applied to test the normal distribution of continuous variables. Differences between anthropometric measurements, body composition, dietary intake, thyroid hormones, lipid profile and physical activity between groups were tested by one-way ANOVA with Post Hoc tests and Kruskal Wallis test, followed by Mann–Whitney U test. Analyses were performed using SPSS version 20 (Chicago, IL, USA). A p value<0.05 was considered statistically significant.

## Results

Anthropometric measurements, body composition and REE in the three groups were shown in Table 1. Waist circumference and BMI were significantly different in two obese groups (p-value < 0.001). There was a significant increase in hip and waist circumference in obese subjects in comparison to normal group (p-value < 0.001). Age and height were similar in three groups. In obese groups fat mass and fat-free mass were significantly increased, however there was no significant difference in body composition between two obese groups (p-value = 0.10, 0.27). Measured resting energy expenditure was significantly decreased in obese with low REE compare to other groups (p-value < 0.001). Moreover measured resting energy expenditure was significantly different between three groups (p-value < 0.001).

**Table 1:** Characteristics of study subjects

Groups/Variables	Obese with low REE N = 16	Obese with normal REE N = 17	P value	Control N = 16	Obese with low REE N = 16	P value	Control N = 16	Obese with normal REE N = 17	P value
Age (years)	37.81 ± 1.64	37.70 ± 1.85	0.99	32.87 ± 1.89	37.81 ± 1.64	0.14	32.87 ± 1.89	37.70 ± 1.85	0.15
Weight (kg)	81.02 ± 2.23	86.86 ± 2.16	0.08	59.38 ± 1.04	81.02 ± 2.23	0.00^*^	59.38 ± 1.04	86.86 ± 2.16	0.00^*^
Height (cm)	161.18 ± 1.40	159.11±1.48	0.32	161.87 ± 1.42	161.18 ± 1.40	0.95	161.87 ± 1.42	159.11 ± 1.48	0.19
BMI (kg/m^2^)	31.23 ±0.68	34.47 ± 0.82	0.00^*^	22.76 ± 0.31	31.23 ±0.68	0.00^*^	22.76 ± 0.31	34.47 ± 0.82	0.00^*^
Waist circumference (cm)	82.75 ± 1.40	90.47 ± 2.16	0.00^**^	67.31 ± 1.08	82.75 ± 1.40	0.00^**^	67.31 ± 1.08	90.47 ± 2.16	0.00^**^
Hip circumference (cm)	111.31 ± 1.95	114.76 ± 2.07	0.37	95.18 ± 1.23	111.31 ± 1.95	0.00^**^	95.18 ± 1.23	114.76 ± 2.07	0.00^**^
REE (kcal/day) Measure	1235.81 ± 30.41	1895.64 ± 76.71	0.00^*^	1624.50 ± 70.82	1235.81 ± 30.41	0.00^*^	1624.50 ± 70.82	1895.64 ± 76.71	0.01^*^
REE (kcal/day) Estimate	1699.78 ± 29.88	1759.39 ± 28.75	0.25	1498.50 ± 19.29	1699.78 ± 29.88	0.00^*^	1498.50 ± 19.29	1759.39 ± 28.75	0.00^*^
FM (kg)	37.08 ± 1.77	41.65 ± 1.46	0.05	18.82 ± 0.54	37.08 ± 1.77	0.00^**^	18.82 ± 0.54	41.65 ± 1.46	0.00^**^
FM (%)	45.43 ± 0.98	47.80 ± 0.71	0.10	31.68 ± 0.69	45.43 ± 0.98	0.00^*^	31.68 ± 0.69	47.80 ± 0.71	0.00^*^
FFM (kg)	43.94 ± 0.64	45.20 ± 0.97	0.52	40.56 ± 0.79	43.94 ± 0.64	0.01^*^	40.56 ± 0.79	45.20 ± 0.97	0.00^*^
FFM (%)	51.56 ± 2.32	52.19 ± 0.71	0.27	68.31 ± 0.69	51.56 ± 2.32	0.00^**^	68.31 ± 0.69	52.19 ± 0.71	0.00^**^

Values are Means ± SE

*Significant difference between REE (Measure), REE (Estimate), FM(%), FFM(kg), weight (kg), BMI(kg/m^2^) by ANOVA with Tukey’s post hoc tests

**Significant difference between FM (kg), FFM (%), REE (Estimate),Waist circumference (cm), Hip circumference (cm) by Kruskal wallis with mann-whitney tests

Table 2 shows dietary intake in three groups. There was no significant difference in energy intake and macronutrients between two obese groups. Moreover there was no significant difference in energy intake and macronutrients between obese groups and normal weight subjects.

**Table 2:** Comparison dietary intake in three groups

Groups/Variables	Obese with low REE N = 16	Obese with normal REE N = 17	P value	Control N = 16	Obese with low REE N = 16	P value	Control N = 16	Obese with normal REE N = 17	P value
Energy intake (kcal/day)	1574.46 ± 108.19	1756.52 ± 136.63	0.32	1773.21 ± 127.51	1574.46 ± 108.19	0.25	1773.21 ± 127.51	1756.52 ± 136.63	0.93
Carbohydrate intake (g/day)	204.93 ± 19.69	220.70 ± 21.20	0.60	213.19 ± 16.89	204.93 ± 19.69	0.31	213.19 ± 16.89	220.70 ± 21.20	0.98
Protein intake (g/day)	62.76 ± 4.36	60.38 ± 5.21	0.74	60.24 ± 4.77	62.76 ± 4.36	0.70	60.24 ± 4.77	60.38 ± 5.21	0.70
Fat intake (g/day)	58.62 ± 5.64	72.61 ± 4.90	0.07	70.56 ± 7.20	58.62 ± 5.64	0.21	70.56 ± 7.20	72.61 ± 4.90	0.81

Values are Means ± SE

Thyroid hormones and lipid profile in three groups were shown in Table 3. There was a significant difference in T_3_ between obese subjects with low REE compared to obese group with normal REE (p-value < 0.001). There was no significant difference in lipid profile between two obese groups. There was a significant difference in LDL, cholesterol and triacylglycerol between obese subjects with low REE compared to normal weight group (p-value < 0.001). Moreover, there was a significant difference in cholesterol and triacylglycerol between obese subjects with normal REE compared to normal weight group (p-value < 0.001).

**Table 3:** Comparison thyroid hormones and lipid profile in three groups

Groups/Variables	Obese with low REE N = 16	Obese with normal REE N = 17	P value	Control N = 16	Obese with low REE N = 16	P value	Control N = 16	Obese with normal REE N = 17	P value
T_3_ (ng/ml)	0.91 ± 0.03	1.13 ± 0.06	0.00^*^	1.06 ± 0.08	0.91 ± 0.03	0.15	1.06 ± 0.08	1.13 ± 0.06	0.14
T_4_ (µg/dl)	8.06± 0.23	7.64 ± 0.47	0.17	7.85 ± 0.66	8.06± 0.23	0.12	7.85 ± 0.66	7.64 ± 0.47	0.82
TSH (mlU/L)	2.16 ± 0.32	2.79 ± 0.74	0.78	2.31 ± 0.43	2.16 ± 0.32	0.94	2.31 ± 0.43	2.79 ± 0.74	0.92
(mg/dl) HDL	41.51 ± 2.95	44.91 ± 3.83	0.95	42.13 ± 2.19	41.51 ± 2.95	0.53	42.13 ± 2.19	44.91 ± 3.83	0.54
(mg/dl) LDL	98.62 ± 4.02	91.76 ± 6.49	0.28	80.93 ± 5.67	98.62 ± 4.02	0.00^**^	80.93 ± 5.67	91.76 ± 6.49	0.13
Total cholesterol (mg/dl)	167.25 ± 3.44	167.46 ± 7.27	0.99	138.87 ± 6.68	167.25 ± 3.44	0.00^*^	138.87 ± 6.68	167.46 ± 7.27	0.00^*^
Triacylglycerol (mg/dl)	135.68 ± 13.42	155.05 ± 22.57	0.47	78.25 ± 7.93	135.68 ± 13.42	0.00^**^	78.25 ± 7.93	155.05 ± 22.57	0.00^**^

Values are Means ± SE

*Significant difference between T_3_, Total cholesterol (mg/dl) by ANOVA with Tukey’s post hoc tests

**Significant difference between LDL (mg/dl), triacylglycerol (mg/dl) by Kruskal wallis with mann-whitney tests

Table 4 shows physical activity according to Met-min/week in three groups. Our finding showed there was no significant difference in physical activity between three groups.

**Table 4:** Comparison physical activity in three groups

Groups/Variables	Obese with low REE N = 16	Obese with normal REE N = 17	P value	Control N = 16	Obese with low REE N = 16	P value	Control N = 16	Obese with normal REE N = 17	P value
Physical activity (Met-min/week)	600 (1290)	720 (2200)	0.62	760 (1875)	600 (1290)	0.46	760 (1875)	720 (2200)	0.89

Median (IQR)

## Discussion

The objectives of this study were to investigate REE differences between obese people, confirm low REE in some obese subjects, and determine differences in dietary, biochemical, anthropometric, and body composition parameters and physical activity in obese women with normal and low resting energy expenditure. We achieved exciting results, although REE was different among obese people in this study, no significant difference was observed in the body composition, physical activity level and dietary intake of these people, so the role of other effective factors in metabolism is raised.

REE is the energy required to maintain body at rest [7]. One of the most effective factors in REE is body composition. Fat-free mass consists of water, protein, and minerals and is the major determinant of REE that explains 60–80% of the inter-individual variance in REE. Fat mass, comprising all body lipids, also contributes to REE variance, especially when subjects with a large range of body mass index (BMI) are investigated [8]. Some studies have showed that since obese subjects have more fat-free mass (FFM) and fat mass (FM) than normal weight subjects, REE is typically higher in obesity [9]. However, other studies such as an investigation by Johannsen et al. have shown that the resting energy expenditure (adjusted for fat- free mass and fat mass) of obese women is similar to lean women [3].

We found a significant difference in the measured REE between two obese groups although there was no difference in their body composition. These results highlight the role of other factors that may be involved in energy metabolism.

Similar to our findings, Rosales-Velderrain et al. reported that the measured resting energy expenditure is much lower than the predicted resting energy expenditure in some obese people [4].

Additionally, a meta-analysis about REE was showed that in obese subjects REE was 3–5% lower than normal weight subjects [10].

Some studies have concluded that increased REE in obesity may be due to obesity-related metabolic risk factors. Insulin resistance and hypertension as a consequence of obesity may lead to a higher REE; these may mask the lower metabolic rate that initially contributes to weight gain [11].

Some mechanisms have been suggested for decreasing REE in obese people. Since mitochondria are essential for energy production at the cellular level, differences in energy expenditure and basal metabolism may be attributed to mitochondrial functions [12].

Similarly, we showed an association between UCP2 protein and resting energy expenditure in another study. We demonstrated an increased risk of reduced resting energy expenditure in obese subjects with low levels of UCP2 protein [13, 14]. Uncoupling proteins (UCPs) are a group of mitochondrial proteins which dissipate proton electrochemical gradient across the mitochondrial membrane. By this mechanism, UCPs may uncouple substrate oxidation from conversion of ADP to ATP, leading to generation of heat and thus increased EE [15]. Moreover, some studies have shown that mitochondrial dysfunction, reduced activity of marker enzymes of oxidative pathways, mitochondrial degeneration, and reduced respiratory capacity in obese people [16, 17]. Furthermore, it has been suggested some adipokines and hormones produced by the adipose tissue may effect the mitochondrial function and energy haemostasis [18].

Some studies have revealed down-regulation of metabolism genes in obese subjects [19]. There are reports of gene repression of fat cell metabolism and decreased fatty acid and triacylglycerol synthesis, insulin-stimulated glucose uptake, and mitochondrial energy metabolism pathways, especially tricarboxylic acid cycle and electron transport chain, in the adipose tissue of obese people [20].

Some other studies have suggested a strong genetic component to REE and have implicated low REE as a predictor of weight gain. Based on these finding, ≥40% of variance in REE (adjusted for age, sex, body mass, and body composition) is estimated to be genetic [17].

In our study, the predicted resting energy expenditure was measured using the Harris-Benedict Eq. [6]. Shaneshin et al. showed the Harris-Benedict formula provides a valid estimate of REE at the group level in a range of normal-weight to morbidly obese Iranians [21].

It is known that body weight depends on the balance between energy intake and energy expenditure in such a way that a positive energy balance results in body mass gain [7]. We noted that energy intake was 300 kcal more than energy expenditure in obese group with low REE; therefore, weight gain is expected. However, in the obese group with normal REE, energy intake was 200 kcal less than energy expenditure, which may refer to under-eating and under-recording of habitual food intake in obese people. It has been shown obese subjects underreport what they consume, specially fat and carbohydrate-rich foods [22]. Moreover, it has been reported that obese people change their food patterns during the recording period [23].

Since diet composition may affect resting energy expenditure and there is no significant difference in dietary intake between obese women with normal REE compared to obese women with low REE, the difference in their REE may not be related to their diet.

We found no significant difference in the lipid profile between obese women with normal and low REE. According to our findings, differences in REE or metabolism in obese subjects are not associated with and do not cause lipid profile changes, although there was a significant difference in lipid profile between obese groups and normal weight subjects.

Our finding showed no difference in physical activity between three groups. Physical activity improves weight loss and is also a good predictor of long-term weight loss maintenance [24]. Some studies have shown that obese women spend significantly more time on resting or sedentary behaviors than lean women every day [25]. Moreover, it has been reported that obese women spend less time being active than lean women, including light, moderate, and vigorous activities. It has been suggested that the minimal amount of energy expenditure by physical activity required for protection against body fat gain is ~ 12 kcal/kg body weight per day [26].

Our findings showed a significant difference in T_3_ between the two obese groups. We believe that the differences in resting energy expenditure between the two obese groups may be related to the level of T_3_. However, the level of thyroid hormones (T_3_, T_4_ and TSH) was within the normal range in all obese subjects.

It should also be considered several limitations of the current study. The first limitation of our study is the small sample size; more studies with more prominent participants are needed. Second, underreporting and under-recording habitual food intakes, typically seen in people with obesity, may contribute to biased findings. However, subjects with extreme dietary intake values were excluded. Another research limitation was possible errors related to measuring REE and body composition due to measurement errors or participant preparation. Despite the abovementioned limitations, this is the first attempt to study external factors such as diet and physical activity and internal factors such as body composition, metabolism, and biochemical factors in participants with different metabolism.

## Conclusions

In conclusion, it was found that REE was much lower in some obese individuals compared to other obese subjects; however, there was no significant difference in their body composition, age, sex, dietary intake, lipid profile and physical activity. Further studies are needed to investigate some factors involved in reduced REE in obese people. Also, more studies are suggested to investigate other factors that affect women’s health and metabolism, such as the status of vitamins and their balance, the use of supplements and other biochemical factors.

## Data Availability

The datasets used and/or analysed during the current study available from the corresponding author on reasonable request.
